# Surface Modification of Cardiovascular Stent Material 316L SS with Estradiol-Loaded Poly (trimethylene carbonate) Film for Better Biocompatibility

**DOI:** 10.3390/polym9110598

**Published:** 2017-11-10

**Authors:** Hang Yao, Jingan Li, Na Li, Kebing Wang, Xin Li, Jin Wang

**Affiliations:** 1 Key Laboratory of Advanced Technology for Materials of Chinese Education Ministry, School of Materials Science and Engineering, Southwest Jiaotong University, Chengdu 610031, China; yaohang1990@my.swjtu.edu.cn (H.Y.); ln1432728994@my.swjtu.edu.cn (N.L.); 2016310129@my.swjtu.edu.cn (K.W.); lixin131715@home.swjtu.edu.cn (X.L.); 2 School of Materials Science and Engineering, Zhengzhou University, Zhengzhou 450000, China

**Keywords:** cardiovascular stent, biocompatibility, surface modification, estradiol, PTMC

## Abstract

A delay in the endothelialization process represents a bottleneck in the application of a drug-eluting stent (DES) during cardiovascular interventional therapy, which may lead to a high risk of late restenosis. In this study, we used a novel active drug, estradiol, which may contribute to surface endothelialization of a DES, and prepared an estradiol-loaded poly (trimethylene carbonate) film (PTMC-E5) on the surface of the DES material, 316L stainless steel (316L SS), in order to evaluate its function in improving surface endothelialization. All the in vitro and in vivo experiments indicated that the PTMC-E5 film significantly improved surface hemocompatibility and anti-hyperplasia, anti-inflammation and pro-endothelialization properties. This novel drug-delivery system may provide a breakthrough for the surface endothelialization of cardiovascular DES.

## 1. Introduction

According to the 2014 global non-communicable diseases status report by World Health Organization (WHO), cardiovascular disease (CVD) is still the leading cause of morbidity and mortality globally (there are 17 million deaths because of CVD worldwide each year, far above the 8 million deaths from cancer) [1]. Cardiovascular interventional therapy with stents has emerged as the most effective method in clinics [2]. However, thrombosis and hyperplasia are the usual pathological responses to the implantation of foreign devices. Although the use of drug-eluting stents (DES) as the mainstream therapeutic interventional implants has been verified owing to effective suppression on the hyperplasia and inflammation [3], the drugs (usually paclitaxel and/or rapamycin) loaded on DES delay vascular healing and the endothelialization process, which may lead to a high risk of late restenosis [4,5]. In the structure of natural blood vessels, a functional endothelium monolayer composed of endothelial cells (EC) maintains the blood vessel patency [6], while the atherosclerosis or stent implantation may damage this functional endothelium monolayer, and further lead to vascular occlusion [7]. Therefore, improving the cardiovascular DES pro-endothelialization property may be an ideal strategy for solving its late restenosis problem.

Surface modification with drug-loaded polymer films has been generally accepted as an effective method for enhancing part of the stent materials’ biocompatibility, and the polymers include polyethylene glycol (PEG) [8], polyacrylamide [9], poly-dopamine (PDA) [10], polyetherimide (PEI) [11] and poly (trimethylene carbonate) (PTMC) [12]. In particular, PTMC is a preferable polymer with a series of superior properties, including non-biological-toxicity, good biocompatibility and biodegradability [13,14]. In addition, it has a certain degree of elasticity and mechanical properties at 37 °C [15], and thus has been widely used in controlled drug release and in vivo implant materials [16,17]. In summary, given all its advantages, PTMC is a potential choice as a drug carrier for surface modification of DES. However, previous research has found that PTMC supported no EC growth and made no contribution to surface endothelialization [18,19]. Therefore, it is paramount to load a pro-endothelial drug on the PTMC film to endow the surface with pro-endothelial property.

For pro-endothelial drugs or factors, there are many choices, such as fibronectin [20], laminin [21], collagen [22], peptides [23] and specific antibodies [24], but most of these drugs or proteins are hydrosoluble, which may lead to initial burst release after loading and implantation, but which is not a favorable durative pro-endothelial property. Estradiol is the most active estrogen that can be secreted by both men and women [25]. It has been proven to contribute to the growth of various cells, such as epithelial, osteocyte and sexual cells [26,27,28], while until now its specific contribution to promoting cardiovascular material surface endothelialization has still not been reported. Most important of all, estradiol is only alcohol soluble [25], which may create a persistent drug release from the PTMC after implantation. Therefore, in this contribution, we make a bold attempt to prepare estradiol-loaded PTMC film (PTMC-E5), place it on to the stainless steel (316L SS) surface of the cardiovascular stent, and evaluate the film’s function in improving 316L SS biocompatibility, including hemocompatibility, anti-hyperplasia, anti-inflammation and pro-endothelializaiton. We sincerely hope this research may provide a point of departure for improving pro-endothelialization of the DES.

## 2. Materials and Methods 

### 2.1 Reagents and Materials

PTMC was purchased from the Chinese Academy of Sciences, Chengdu Organic Chemistry Co., Ltd., Chengdu, China. The estradiol was received from Meilunbio Co., Ltd., Dalian, China. All of the reagents used in our study were of analytical grade. Demineralized water (dH_2_O) was used in the experiments. 

### 2.2 Preparation of PTMC-E5 Film

The 316L stainless steel (316L SS) plates (Northwest Institute for Non-ferrous Metal Research, Baoji, China) were cut into small discs of 10 mm diameter and polished. Then, the 316L SS disks were sonicated successively in acetone, ethanol, and deionized water, and finally dried at room temperature. The clear 316L SS were immersed into a mixed solution composed of PTMC (3.33 mg/mL) and estradiol (5%) (the solvent was composed of dichloromethane and ethyl acetate with a volume ratio of 2:1), and then incubated in a draught cupboard at room temperature for 24 h to vaporize the solvent. The film obtained on the bare 316L SS was labeled as PTMC-E5, and the control film without estradiol was labeled as PTMC.

### 2.3 Surface Characterization of PTMC-E5 Film

The morphologies of bare 316L SS, PTMC and PTMC-E5 samples were observed by scanning electron microscopy (SEM, JSM-7001F, Japan Electron Optics Laboratory Co., Ltd., Tokyo, Japan), and the surface chemical composition of PTMC-E5 was examined using Fourier transform infrared spectrometry (FTIR, NICOLET 5700, Thermo Electron Corporation, Waltham, MA, USA) with reflectance mode and X-ray photoelectron spectroscopy (XPS, K-Alpha, Thermo Electron Limited, Winsford, UK) [29,30,31]. The wettability of the bare 316L SS, PTMC and PTMC-E5 sample surfaces was assessed by water and blood contact angle measurement (DSA 100, Krüss GmbH, Hamburg, Germany) [32]: the samples were first dried and then fixed to a glass slide. A droplet of dH_2_O was added to the surface to detect contact angle using a horizontal microscope. For each sample, the mean value of the contact angle was calculated from at least three individual measurements taken at different locations on the samples examined. The drug-eluting portion of the estradiol was also investigated, as described elsewhere [16].

### 2.4 Platelet Adhesion Test of PTMC-E5 Film

The fresh, human, whole blood used in this experiment was obtained legally from the central blood station of Chengdu, China. The analysis was performed within 2 h of the blood donation. Platelet-rich plasma (PRP) was prepared by centrifuging (1500 rpm, 15 min) the fresh, human, whole blood; and 50 μL of fresh PRP was distributed on the samples and incubated for 1 h at 37 °C. After washing with normal saline (NS), the samples were fixed for 2 h using 2.5% glutaraldehyde solution at 25 °C, then washed again with NS three times, and subsequently stained by rhodamine (100 μg/mL, Sigma, Ontario, CA, USA) for 15 min. After a washing step, the morphology of the adherent platelets was observed by inverted fluorescence optical microscopy (OLIMPUS-IX51, Olympus Ltd., Tokyo, Japan) [33]. Comparative analysis of the adherent and activated platelets was performed in order to evaluate the hemocompatibility of 316L SS, PTMC and PTMC-E5, respectively [34]. A dynamic whole-blood experiment was also performed for 1 h to simulate the interaction of blood flow and the surfaces in vivo [10].

### 2.5 Smooth Muscle Cells Culture 

Smooth muscle cells derived from the human umbilical artery (HUASMC) were isolated and cultured using the following method [35]: the human umbilical cord was washed thoroughly with NS to remove the blood outside, and then the artery was excised from the umbilical cord and opened at its length. The external connective tissue and fibroblast layer were peeled off. The endothelial cells inside were gently scraped using a sharp tweezer. The muscle tissue was washed thoroughly with NS and cut into small fragments. The fragments were then seeded in a single-used culture flask filled with 4 mL medium F12 and 1 mL fetal calf serum (FCS, Gibco BRL, Gaithersburg, MD, USA) mixture, and incubated at 37 °C in a humidified atmosphere containing 95% air and 5% CO_2_. The fragments were removed after HUASMC migrated to the culture flask. Replicated cultures were performed by trypsinization when cells were approaching confluence. Cells were fed with freshly prepared growth medium every 24 h. The 3rd passage of HUASMC was used to evaluate the anti-hyperplasia of PTMC-E5 film.

In brief, the HUASMC was seeded on to surfaces of 316L SS, PTMC and PTMC-E5, and incubated in the standard condition above for 1 day and 3 days, respectively. Then, the samples were picked up and washed with NS at 37 °C three times, and subsequently fixed with 4% paraformaldehyde (Sigma, Ontario, CA, USA) for 30 min at 25 °C. After a rinsed step, the HUASMC on each sample was stained with the rhodamine reagent for 15 min and observed under inverted fluorescence microscopy (OLIMPUS-IX51, Japan) [36]. The HUASMC numbers of 316L SS, PTMC and PTMC-E5 were determined via a typical CCK-8 assay [36].

### 2.6 Endothelial Cells Culture

Human umbilical vein endothelial cells (HUVEC) obtained from the newborn umbilical cord (Huaxi Hospital, Chengdu, China) were cultured in a humidified incubator with 95% air and 5% CO_2_. HUVEC between the 3rd and 5th passages were used for experiments. The 316L SS, PTMC and PTMC-E5 samples were placed in a 24-well culture plate, and the HUVEC were seeded on to the samples with the concentration of 5 × 10^4^ cells/mL, then cultured at 37 °C for 4 h, 1 day and 3 days, respectively. After the sequentially washed step, the samples were fixed with 4% paraformaldehyde (Sigma, USA) for 2 h at room temperature and stained by a rhodamine reagent (Sigma, Ontario, CA, USA) for 15 min, then finally examined and recorded by a fluorescence microscope (DMRX, Leica, Solms, Germany). A CCK-8 assay was performed to investigate the HUVEC attachment and proliferation on the samples [37].

### 2.7 In Vivo Tissue-Response Test of PTMC-E5 Film

The study was conducted in accordance with relevant national legislation on the use of animals for research and the protocol was approved by the Ethics Committee of Sichuan province and Southwest Jiaotong University (Project identification code: SYXK(Chuan)2014-189). This experiment was carried out by implanting the bare 316L SS wires (Φ 0.1 mm × 10 mm) and PTMC-E5 coated 316L SS wires in the lumen of SD rats’ abdominal aorta for 1 month [31]. In brief, the wire remained in contact with flowing blood within the aorta to simulate the presence of a stent strut, with some regions of the wire in direct contact with the arterial wall and some not in contact. After 1 month, the aortas containing the implanted wires were harvested for histological analysis. Rat aortas containing the 316L SS wire implants were snap-frozen in liquid nitrogen and cryo-sectioned for histological analysis. Cross sections were ethanol-fixed and then stained with antibodies specific for EC (CD31, Sigma, Ontario, CA, USA), Smooth muscle cells (SMC, α-SMA, Sigma, Ontario, CA, USA) and macrophages (TNF-α, Sigma, Ontario, CA, USA). The nuclei of all the cells were stained by 4,6-diamino-2-phenyl indole (DAPI, Sigma, Ontario, CA, USA). The stained images were observed by confocal laser-scanning microscopy (CLSM, Nikon Eclipse Ti, Nikon, Tokyo, Japan).

### 2.8 Statistical Analysis

Mean values ± SD are given with their representative images. Statistical significance requires a *p*-value < 0.05.

## 3. Results and Discussion

### 3.1. Surface Characterization

**Figure 1:**
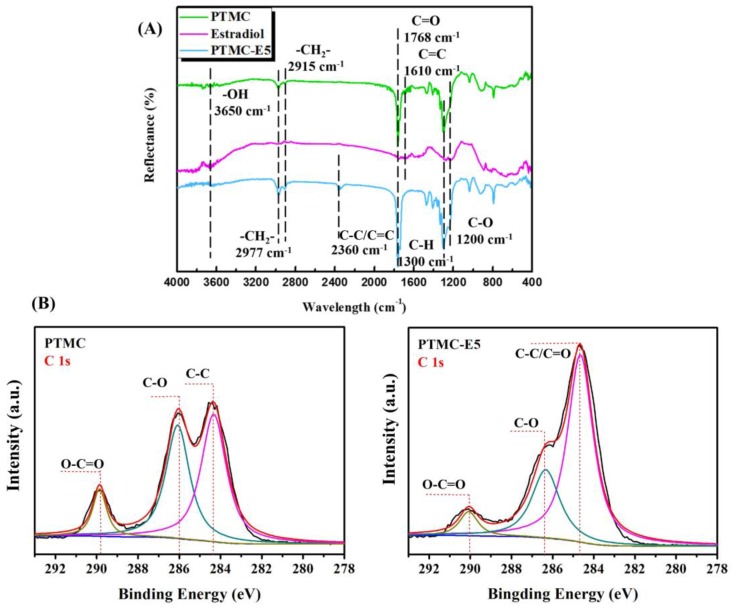
(**A**) Attenuated total reflection Fourier transform infrared spectrometry (ATR-FTIR) and (**B**) X-ray photoelectron spectroscopy (XPS) spectra of PTMC, estradiol and PTMC-E5.

To confirm the immobilization of PTMC-E5 film on to the 316L SS, attenuated total reflection FTIR (ATR-FTIR) spectroscopy was undertaken in order to characterize changes in chemical structure, and the results are displayed in Figure 1 A. As shown in Figure 1A, the PTMC, estradiol controls and PTMC-E5 film had a broad peak around 3650 cm^−1^ ascribed to –OH stretching vibrations, and the peaks at 1768 cm^−1^ and 1300 cm^−1^ were ascribed to C=O and C–H stretching vibrations, respectively. The peaks at 2977 cm^−1^ and 2915 cm^−1^ corresponded to the –CH_2_– group, suggesting the existence of PTMC in the PTMC-E5 film. The peaks appearing at 1610 cm^−1^ corresponded to C=C stretching vibrations of both PTMC and estradiol, but disappeared in the PTMC-E5 film, which may be due to the vibration cancellation. The stretching vibrations of C–O in the PTMC located at 1200 cm^−1^, but shifted to the higher wavelength; thus, the further shift of C–O to the higher wavelength in the PTMC-E5 coating indicated the existence of both PTMC and estradiol indirectly. A new peak around 2360 cm^−1^ may have been caused by the stretching vibrations of the C–C and C=C bonds, which were influenced by the preparation of PTMC-E5 film. Further comparison of the C 1s high-resolution spectra in Figure 1B confirmed the ATR-FTIR result. On the PTMC surface, the peaks at 286.82 and 288.81 eV correlated to the carbon in C–O and the carboxyl carbon in O–C=O, respectively, and the peak at 284.73 eV was attributed to the presence of C–C. On the PTMC-E5 surface, the peak of estradiol C=C appeared at 284.5 eV, and a large peak was synthesized in the spectrum because the peak values of C=C and C–C bonds were very close. In this surface, the peaks at 284.5 eV were significantly increased, and the shifted peaks of C–O and O–C=O were weaker due to group stacking. All the results above indicated the successful preparation of PTMC-E5 film on to the 316L SS.

**Figure 2:**
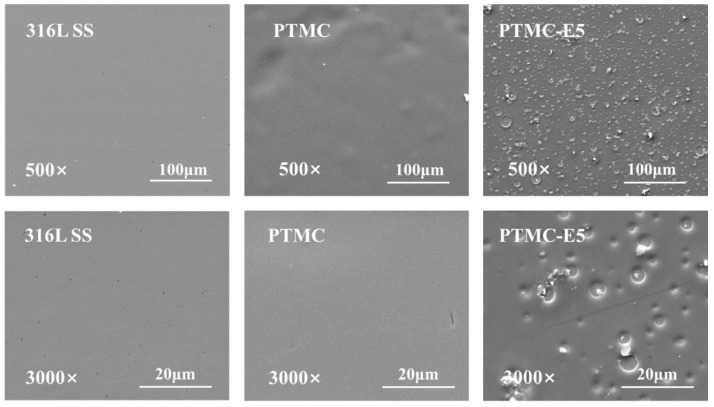
Scanning electron microscopy (SEM) images of 316L SS, PTMC and PTMC-E5.

Figure 2 shows the surface morphology changes on the 316L SS, PTMC film and PTMC-E5 film. The 316L SS substrate showed an obvious smoother surface after the PTMC coating preparation, while a porous surface appeared after the PTMC-E5 film preparation. The scales of the voids ranged from several micro-meters to nano-meters, and this will affect cell behaviors on the surface, such as adhesion, proliferation and distribution [38,39].

**Figure 3:**
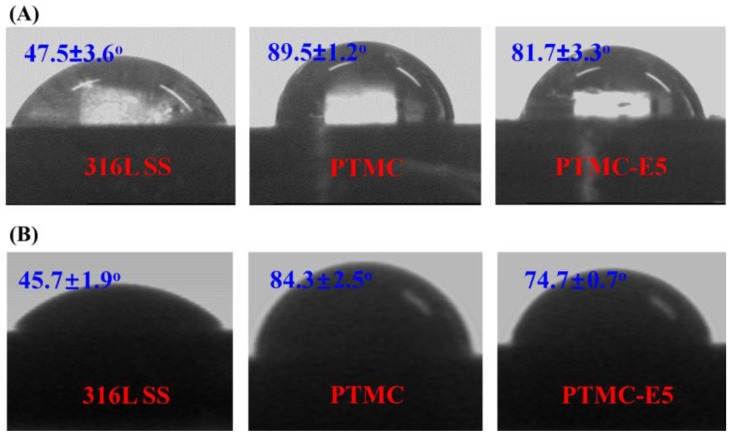
(**A**) Water and (**B**) blood contact angles of 316L SS, PTMC and PTMC-E5. (mean ± SD, *n* = 6).

Surface hydrophilicity may influence the biocompatibility of the materials by causing quantitative and qualitative variation in the adsorbed protein [40]. In this work, the water and blood contact angles were detected to evaluate the surface hydrophilicity of PTMC-E5 film. Figure 3A showed the water contact angle of 316L SS, PTMC-coated 316L SS and PTMC-E5 coated 316L SS. The water contact angle significantly increased after the PTMC film preparation, indicating a relatively hydrophobic surface, which was attributed to its high molecular weight and methylene group. However, after the estradiol loaded, the water contact angle decreased due to the introduction of its hydroxyl group and the changed roughness, which indicated higher hydrophilicity, which would contribute to protein absorption and further EC adhesion/proliferation. The blood contact angle showed a consistent trend with the water contact angle, but the blood values seemed a little smaller than the water values on each sample (Figure 3B), which may be due to the proteins, platelets and other cells.

**Figure 4:**
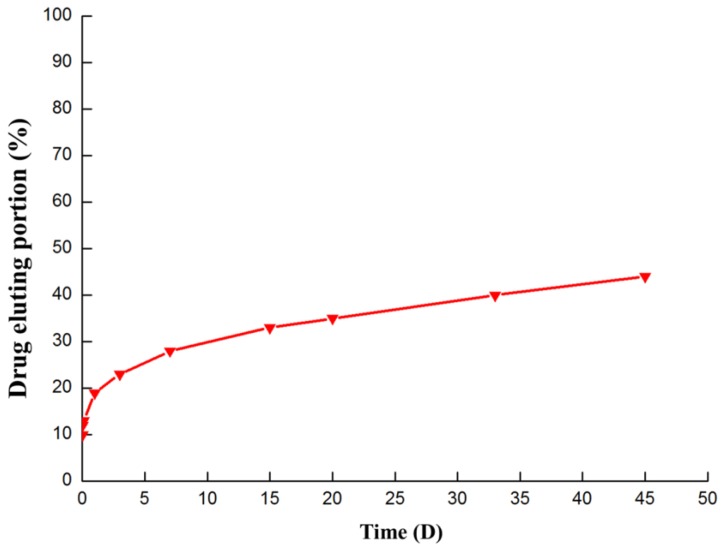
Drug-eluting portion of estradiol from the PTMC-E5 samples.

The estradiol eluting from the PTMC-E5 samples under a 15 dyn/cm^2^ fluid flow (dH_2_O) was investigated in order to evaluate the stability of PTMC-E5. Figure 4 shows that there was a burst release at the beginning, which may have been due to the physically attached estradiol falling off. Then, the eluting curve gradually leveled off, suggesting slow-release of the estradiol. Until 45 days later, there was still estradiol eluted from the PTMC-E5 film. All the results indicated that the PTMC-E5 film possessed good stability and a delayed release ability.

### 3.2. Biocompatibility of PTMC-E5 Film

**Figure 5:**
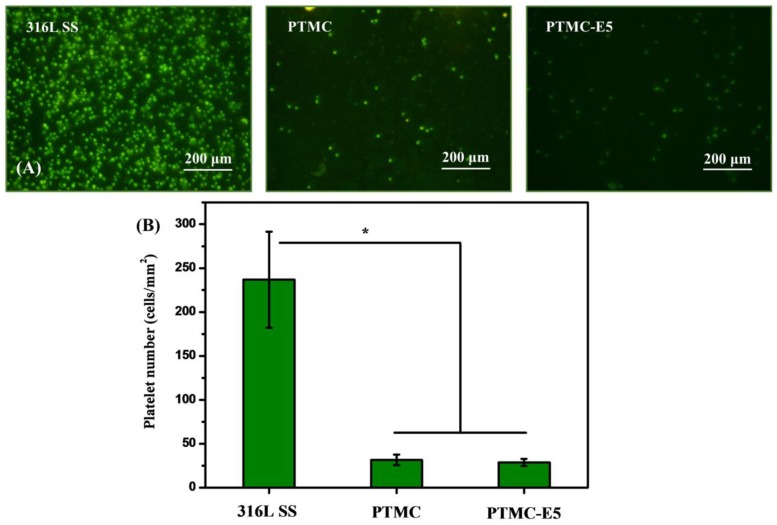
(**A**) Fluorescence-staining and (**B**) quantitative characterization using lactate dehydrogenase serum (LDH) of adherent platelets on the 316L SS, PTMC and PTMC-E5 samples. (mean ± SD, * *p* < 0.05, *n* = 3).

**Figure 6:**
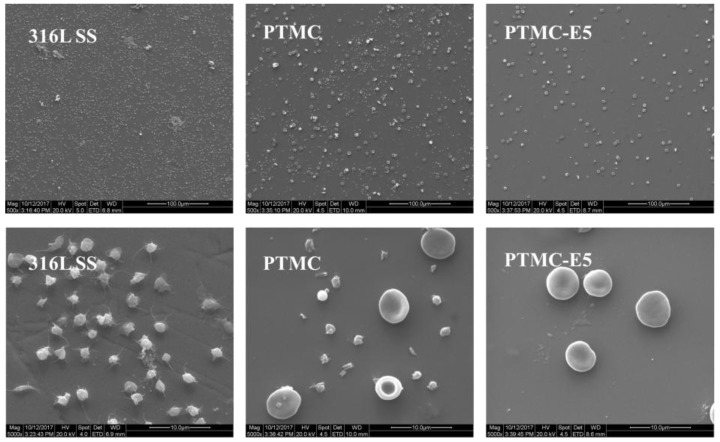
SEM images of the blood components on the 316L SS, PTMC and PTMC-E5 samples after the action of a 15 dyn/cm^2^ blood flow for 1 h.

The adhesion and aggregation of platelets on stent surfaces can lead to coagulation and thrombosis [41]. Therefore, the in vitro platelet adhesion test is often applied to evaluate the hemocompatibility of the stent material’s surface. Fluorescence-staining images of the rhodamine-stained platelets on each sample were presented in Figure 5A, and this result showed that there were fewer platelets adhered to the PTMC and PTMC-E5 compared with the 316L SS surface, while the platelets on the 316L SS seemed more aggregated than those on the PTMC and PTMC-E5 films. The quantitative characterization of adherent platelets by a typical Lactate dehydrogenase (LDH) method presented consistent results: PTMC and PTMC-E5 < 316L SS (Figure 5B), which indicated better hemocompatibility. To simulate blood flow acting on the surfaces, we further performed a dynastic whole-blood experiment (15 dyn/cm^2^) to investigate the overall hemocompatibility of the PTMC-E5 film. Figure 6 shows that there were still numerous activated platelets and little red blood cells (RBC) on the 316L SS surface, and the PTMC film obviously suppressed platelet adhesion and activation, but several numbers of adherent platelets could still be seen. It is surprising that the PTMC-E5 film showed a much smaller platelet number compared with the PTMC and 316L SS, suggesting excellent hemocompatibility, while the application of E5 as an anti-coagulant drug has not been demonstrated. Another novel finding was that the porous structures on the PTMC-E5 film disappeared after the action of the blood flow, and this may be attributed to the blocking of proteins (such as albumin) in the blood flow.

**Figure 7:**
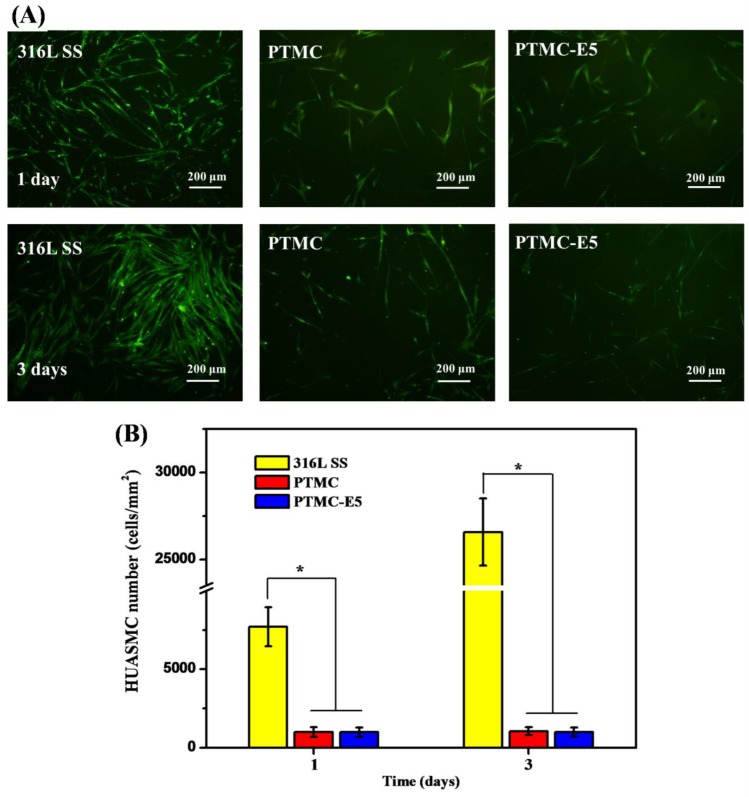
(**A**) Fluorescence images and (**B**) quantitative characterization of smooth muscle cells derived from the human umbilical artery (HUASMCs) on samples of 316L SS, PTMC and PTMC-E5, respectively (mean ± SD, * *p* < 0.05, *n* = 3).

After the stent intervention, the smooth muscle cells located at the blood vessel media will be influenced by the damaged vessel wall, change their phenotype from contractile to synthetic, pathologically proliferate and migrate on to the stents’ surfaces, which will lead to in-stent restenosis thereby creating a key challenge for the long-term therapy [42,43]. Thus, HUASMC adhesion and proliferation were investigated in order to evaluate the anti-restenosis ability of each surface. The morphology analysis (Figure 7A) and adhesion/proliferation determination (Figure 7B) of the HUASMCs indicated that the PTMC and PTMC-E5 film significantly reduced HUASMC quantity on the 316L SS, suggesting suppression of HUASMC adhesion/proliferation and further hyperplasia.

**Figure 8:**
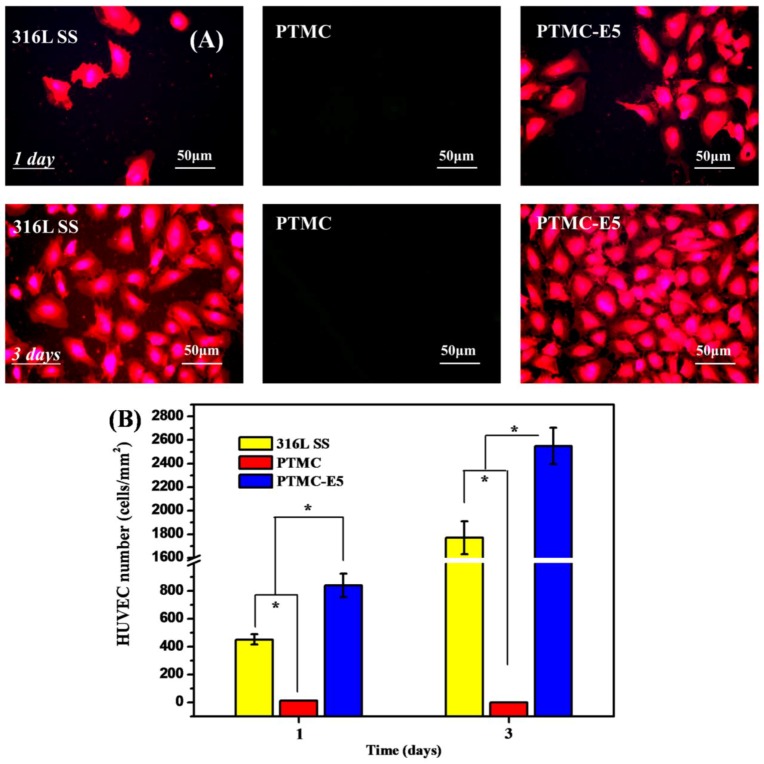
(**A**) Fluorescence images and (**B**) quantitative characterization of human umbilical vein endothelial cells (HUVEC) on samples of 316L SS, PTMC and PTMC-E5, respectively. (mean ± SD, * *p* < 0.05, *n* = 3).

To detect the adhesion and proliferation of endothelial cells on 316L SS, PTMC and PTMC-E5, HUVEC were seeded on the surface of different samples. After culture for 1 day and 3 days, the morphology and behavior of the HUVEC was obsverved by fluorescence images in Figure 8A. The HUVEC on both 316L SS and PTMC-E5 presented elliptical, spherical, or polygonal morphology, but there seemed to be no HUVEC on the PTMC surface, which indicated a strongly inhibiting effect of PTMC on HUVEC growth. The HUVEC number on each sample showed a trend of PTMC-E5 > 316L SS > PTMC. The CCK-8 determination in Figure 8B showed consistent results, and all the in vitro results indicated that the PTMC-E5 film could significantly improve the endothelialization of 316L SS and PTMC. It is worth noting that surface roughness and morphology are important factors that will influence HUVEC behavior; our previous study has proved that the surface micro/nano structures significantly improve endothelial cell proliferation [33,39]. Thus, voids ranging from nano-meters to micro-meters on the PTMC-E5 surface made a contribution to surface endothelialization.

**Figure 9:**
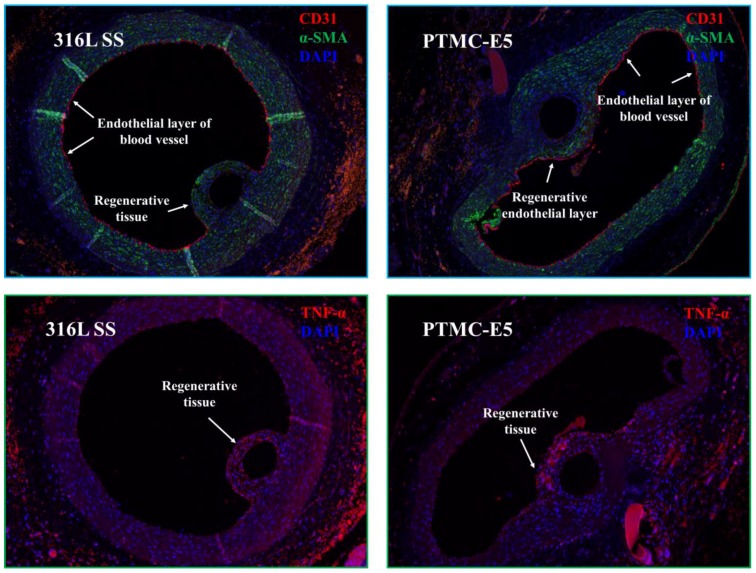
TNF-α, α-SMA and CD31 staining for artery tissues around PTMC-E5 and 316L SS implants after 1 month (the nuclei were stained with DAPI, labeled with blue).

To further investigate the cell−material interaction of the PTMC-E5 film in vivo, the bare 316L SS samples and PTMC-E5 coated 316L SS samples were implanted in the aortic implantation of SD rats for 1 month. Figure 9 shows that there were significantly less TNF-α expression on the regenerative tissues around the PTMC-E5 samples compared to that around the 316L SS samples, suggesting slighter inflammation. In addition, it was obvious that the regenerative tissues around both the PTMC-E5 and 316L SS samples showed positive α-SMA expression, suggesting a contractile phenotype of these smooth muscle cells. Tissues close to the blood flow and endothelial layer showed more positive α-SMA expression, which indicated that the phenotypes of these smooth muscle cells was regulated by the blood flowing, and contractile smooth muscle cells were necessary for the regeneration of the endothelial layer. The CD31 staining showed that the PTMC-E5 samples possessed a relatively complete surrounding endothelial monolayer, indicating preliminary surface endothelialization. There was little positive expression of CD31 around the 316L SS samples, and this endothelial layer seemed to be incomplete. Therefore, the CD31 staining results indicated that the PTMC-E5 film significantly promoted surface endothelialization of 316L SS.

## 4. Conclusions

In this work, we designed a novel PTMC-E5 film on the cardiovascular stent material 316L SS. The characterization of FTIR, SEM and water/blood contact angle measurement proved that the PTMC-E5 film had been successfully prepared. The results of tests on the drug elution of estradiol demonstrated the PTMC-E5 film’s stability. Platelet adhesion and dynastic whole-blood experiment results demonstrated the improved hemocompatibility of the 316L SS by PTMC-E5 modification, and a HUASMC culture experiment presented a consistent result that both the PTMC and PTMC-E5 films inhibited smooth muscle-cell adhesion and proliferation, suggesting a strong anti-hyperplasia function. In particular, the PTMC-E5 film exhibited an excellent ability to improve endothelial cell adhesion and proliferation, indicating a potential function of pro-endothelialization. In vivo test in SD rats’ coeliac arteries further verified this: compared with the bare 316L SS wire, the PTMC-E5 coated 316L SS wire obviously improved endothelial layer regeneration (CD31 positive expression). All the results revealed that this PTMC-E5 film could markedly improve surface biocompatibility and, in particular, promote endothelial layer regeneration, and thus has potential applications for the surface modification of cardiovascular biomaterials.
